# Hemodynamic Responses to Exercise and Predictors of Exercise Capacity in Mitral Stenosis: Exercise Catheterization Study

**DOI:** 10.1016/j.jacasi.2026.04.040

**Published:** 2026-06-25

**Authors:** Jihoon Kim, Ki Hong Choi, Jiwon Kim, Jihee Son, Eun Kyoung Kim, Sung-A Chang, Sang-Chol Lee, Seung Woo Park, Sung-Ji Park, Jeong Hoon Yang

**Affiliations:** a Division of Cardiology, Department of Internal Medicine, Heart Vascular Stroke Institute, Samsung Medical Center, Sungkyunkwan University School of Medicine, Seoul, South Korea; b Department of Critical Care Medicine, Samsung Medical Center, Sungkyunkwan University School of Medicine, Seoul, South Korea

**Keywords:** catheterization, echocardiography, exercise, mitral stenosis

## Abstract

**Background:**

Although a mitral valve area (MVA) of <1.5 cm^2^ is a major indication for intervention in mitral stenosis (MS), patients in this group may exhibit wide variability in exercise tolerance.

**Objectives:**

The aim of this study was to assess hemodynamic changes during exercise in patients with severe MS using exercise cardiac catheterization.

**Methods:**

This prospective study enrolled 30 patients with MVA <1.5 cm^2^ who underwent exercise catheterization using a supine bicycle between 2021 and 2024. Measurements from 30 MS patients were compared with those from 17 control participants without evidence of heart failure (noncardiac dyspnea [NCD] group). Exercise capacity was quantified by peak oxygen consumption (Vo_2_).

**Results:**

At peak exercise, mean pulmonary artery wedge pressure was markedly higher in the MS group. Peak Vo_2_ was significantly lower in MS than in NCD patients (14.2 mL/kg/min vs 16.5 mL/kg/min; *P* = 0.027), while the arteriovenous oxygen difference was higher in the MS group (9.6 mL/dL vs 7.1 mL/dL; *P* < 0.001). Although the cardiac index did not significantly differ between the 2 groups at rest, it was significantly lower in the MS group at peak exercise (5.7 L/min/m^2^ vs 9.4 L/min/m^2^; *P* < 0.001). In the MS group, the parameters associated with peak Vo_2_ were left ventricular global longitudinal strain, stroke volume index, and MVA, as calculated by the continuity equation, but not planimetry MVA or pressure gradient.

**Conclusions:**

Patients with MVA <1.5 cm^2^ exhibited limited exercise tolerance compared with those with NCD. Left ventricular global longitudinal strain and resting stroke volume index emerged as key parameters associated with exercise tolerance among MS patients.

Mitral stenosis (MS) is a progressive heart valve disease characterized by the narrowing of the mitral valve (MV) opening, which impedes normal blood flow from the left atrium (LA) to the left ventricle (LV) during diastole. Consequently, patients with MS commonly present with symptoms such as fatigue and dyspnea on exertion, leading to exercise intolerance. Previous studies have demonstrated that chronotropic incompetence and peripheral factors are major determinants of exercise intolerance in MS, whereas impaired valvular function, such as MV area (MVA) or pressure gradient, is not.^1^, ^2^, ^3^ These findings underscore the value of stress testing in revealing symptoms and evaluating physiological adaptation to exercise in patients with mild or inconsistent findings at rest.^4^

Current guidelines recommend MVA ≤1.5 cm^2^ as a Class 1 indication for intervention in symptomatic patients with rheumatic MS.^5^^,^^6^ However, symptoms are often subjective, and exercise intolerance can be influenced by extravalvular factors, not simply by MVA.^2^ Consequently, there is often a mismatch between symptoms and anatomical severity in this patient group. To date, no resting parameters have been clearly shown to correlate with exercise capacity, highlighting the need for a better understanding of the mechanisms underlying exercise intolerance in patients with MS. Consequently, discrepancies between symptoms and disease severity often complicate clinical decision-making in this heterogeneous patient group. Therefore, we conducted a prospective study to assess hemodynamic responses to exercise and identify predictors of exercise intolerance in patients with MVA ≤1.5 cm^2^.

## Methods

### Study population and data collection

This was a prospective, single-center observational study conducted from September 2021 to December 2024 at Samsung Medical Center in the Republic of Korea. Consecutive patients with moderate to severe MS who were scheduled for exercise cardiac catheterization were enrolled. The inclusion criteria were age >19 years, rheumatic MS, and MVA ≤1.5 cm^2^, measured using 2-dimensional (2D) planimetry, without moderate or severe mitral regurgitation or other significant valvular diseases. Clinical and laboratory data were collected from the medical records and patient interviews. The noncardiac dyspnea (NCD) group consisted of patients undergoing exercise catheterization who had no evidence of heart failure according to the following criteria: 1) resting pulmonary artery wedge pressure (PAWP) <15 mm Hg; 2) peak exercise PAWP <25 mm Hg; 3) PAWP/cardiac output (CO) slope <2 mm Hg/L/min; and 4) mean pulmonary artery pressure (PAP)/CO slope <3 mm Hg/L/min.^7^^,^^8^ The reason for exercise testing in the NCD group was dyspnea on exertion or equivalent symptoms in the absence of a documented cause. All participants included in this study had a preserved LV ejection fractions of 50% or higher, and patients with significant cardiopulmonary conditions that could affect symptoms or exercise capacity, such as chronic lung disease or coronary artery disease, were not included. The study protocol was approved by the Institutional Review Board of Samsung Medical Center, and informed consent was obtained before catheterization (2020-03-013). This study was conducted in accordance with the Declaration of Helsinki.

### Transthoracic echocardiography

Echocardiography was performed using a Vivid 7 ultrasound machine (GE Medical Systems). Standard 2D echocardiographic measurements were conducted in accordance with the recommendations.^9^^,^^10^ Stroke volume was calculated using the LV outflow tract diameter and the time-velocity integral of pulsed-wave Doppler and was indexed to body surface area (stroke volume index). MVA was measured on 2D planimetry from the parasternal short-axis view, selecting the best orifice cross-sectional plane. Additionally, MVA was calculated using the pressure half-time (PHT) method or the continuity equation (CE) on the basis of stroke volume and the time-velocity integral of continuous-wave Doppler of mitral inflow. Transmitral mean diastolic pressure gradient (MDPG) was measured from continuous-wave Doppler.

For strain measurements, echocardiographic images were digitally stored and analyzed off-line using vendor-independent postprocessing software (2D Cardiac Performance Analysis version 1.4, TomTec Imaging Systems). Analyses were performed by an experienced cardiologist who was blinded to clinical information. The LV endocardial borders were traced on the end-systolic and end-diastolic frames in the apical 4-, 2-, and 3-chamber views.^11^ The LA endocardial borders were traced on the end-systolic and end-diastolic frames from a nonforeshortened apical 4-chamber view.^12^ LV global longitudinal strain (GLS) and LA reservoir strain were automatically calculated as the average of segmental longitudinal endocardial strain values at the end-systolic phase. For LV GLS, the absolute values were used for comparison. In patients with atrial fibrillation, echocardiographic parameters were averaged over 3 consecutive beats with a heart rate of 60 to 100 beats/min.

### Cardiac catheterization and exercise protocol

Beta-blockers and nondihydropyridine calcium-channel blockers were discontinued at least 3 days before catheterization. Cardiac catheterization and maximal-effort supine bicycle exercise with simultaneous echocardiography, expired gas analysis, and blood gas analysis were performed using methods that our group previously described.^13^

Right heart catheterization was performed without sedation using a Swan-Ganz catheter via the right internal jugular vein by experienced cardiologists (K.H.C. and J.H.Y.). The transducer was positioned at the midchest level, corresponding to the position of the LA in the supine patient. Proper zeroing was performed before the preparation and initiation of the procedure. Pressure profiles, including PAWP, systolic, diastolic, and mean PAP, and right atrial pressure, were recorded as the mean of ≥3 consecutive beats at end-expiration.^14^ Pressure tracings were digitized (240 Hz) and stored for off-line analysis. Arterial blood gas analyses were performed using samples obtained via an indwelling arterial line, and venous blood gas analyses were conducted on samples from the pulmonary artery and right atrium. The arteriovenous oxygen difference was calculated using directly measured arterial and mixed venous oxygen content from blood samples. Breath-by-breath minute ventilation, carbon dioxide production, and oxygen consumption (Vo_2_) were measured. CO was calculated using the direct Fick method (CO = Vo_2_/arteriovenous O_2_ difference) and indexed to body surface area. Pulmonary vascular resistance was calculated as: (mean PAP − PAWP)/cardiac output (not stroke volume), stroke volume was derived as: CO/heart rate, and pulmonary arterial compliance was calculated as stroke volume/pulmonary pulse pressure. Ventilatory efficiency was assessed using the minute ventilation/carbon dioxide production slope. For patients with MS, a pigtail catheter was maintained in the LV to assess LV end-diastolic pressure and transmitral MDPG at rest and during exercise.

Following baseline measurements, patients performed supine cycle ergometry, starting at 20 W of work load with 10- to 20-W increments every 3 minutes until subject-reported exhaustion. Hemodynamic data were acquired at 20 W and peak exercise using the same methods across all participants.

### Statistical analysis

Categorical variables are presented as frequencies and percentages, and continuous variables are presented as mean ± SD for consistency of presentation. Between-group differences were compared using a chi-square test for categorical variables and either Student’s *t*-test or the Mann-Whitney *U* test for continuous variables, depending on the normality of their distribution assessed using the Shapiro-Wilk test. Correlations were assessed using Pearson’s correlation coefficient.

Exercise hemodynamic status of the MS group were compared with the NCD group at rest, 20 W, and peak exercise. In the MS group, univariable linear regression analysis was performed to identify parameters associated with peak Vo_2_ among rest and exercise parameters, as well as to assess correlations between LV GLS and exercise hemodynamic parameters. To examine the independent association of LV GLS with peak Vo_2_, a multivariable linear regression model was constructed with adjustment for key clinically relevant covariates: sex and atrial fibrillation. All tests were 2-sided, and a *P* value <0.05 was considered to indicate statistical significance. Statistical analysis was performed using R version 3.6.3 (R Foundation for Statistical Computing).

## Results

### Baseline characteristics

The present analysis included 30 patients with MS and 17 NCD patients. The median age was not significantly different between the 2 groups, but the MS group had a lower proportion of men (23.3% vs 58.8%; *P* = 0.034) (Table 1). In the MS group, the prevalence of atrial fibrillation was markedly higher (43.3% vs 0%; *P* = 0.004), and N-terminal pro–B-type natriuretic peptide levels were significantly elevated (mean 569.4 pg/mL vs 45.1 pg/mL; *P* < 0.001).

**Table 1:** Baseline Clinical and Echocardiographic Parameters

	NCD (n = 17)	MS (n = 30)	*P* Value
Demographics			
Age, y	53.9 ± 14.0	57.0 ± 8.9	0.690
Male	10 (58.8)	7 (23.3)	**0.034**
Height, cm	165.8 ± 7.4	160.3 ± 6.9	**0.014**
Weight, kg	67.6 ± 9.3	60.2 ± 9.3	**0.012**
Body mass index, kg/m^2^	24.2 ± 3.0	23.1 ± 2.7	0.208
Body surface area, m^2^	1.8 ± 0.1	1.6 ± 0.1	**0.004**
NYHA functional class			**0.010**
I	8 (47.1)	5 (16.7)	
II	9 (52.9)	15 (50.0)	
III	0 (0)	10 (33.3)	
Comorbidities and laboratory values			
Hypertension	8 (40.0)	7 (23.3)	0.588
Diabetes	4 (20.0)	3 (10.0)	0.409
Atrial fibrillation	0 (0)	13 (43.3)	**0.004**
Hemoglobin, g/dL	13.7 ± 1.2	13.1 ± 1.2	0.103
NT-proBNP, pg/mL	45.1 ± 33.9	569.4 ± 559.1	**<0.001**
Echocardiography			
LVIDD, mm	50.7 ± 4.1	48.0 ± 3.7	**0.026**
LVISD, mm	30.9 ± 3.5	29.9 ± 3.4	0.321
LVEF, %	63.5 ± 5.2	61.3 ± 4.4	**0.138**
E′, cm/s	8.0 ± 3.0	5.7 ± 1.8	**0.007**
LA diameter, mm	35.9 ± 5.9	52.7 ± 7.2	**<0.001**
LA volume index, mL/m^2^	32.6 ± 7.0	76.0 ± 24.5	**<0.001**
RVSP, mm Hg	25.4 ± 5.9	35.0 ± 10.4	**<0.001**
Mitral valve			
Wilkins score		8.6 ± 1.2	
2D MVA, cm^2^	—	1.1 ± 0.2	—
<1.0	—	7 (23.3)	—
≥1.0 and <1.5	—	23 (76.7)	—
PHT MVA, cm^2^	—	1.2 ± 0.3	—
CE MVA, cm^2^	—	1.0 ± 0.3	—
Transmitral MDPG, mm Hg	—	9.8 ± 5.1	—

Values are mean ± SD or n (%). *P* values in **bold** denote statistical significance.

2D = 2-dimensional; CE = continuity equation; LA = left atrial; LVEF = left ventricular ejection fraction; LVIDD = left ventricular internal diastolic dimension; LVISD = left ventricular internal systolic dimension; MDPG = mean diastolic pressure gradient; MS = mitral stenosis; MVA = mitral valve area; NCD = noncardiac dyspnea; NT-proBNP = N-terminal pro–B-type natriuretic peptide; PG = pressure gradient; PHT = pressure half-time; RVSP = right ventricular systolic pressure.

Baseline echocardiography revealed lower LV end-diastolic dimensions and LV ejection fractions, along with larger LA diameters and a higher LA volume indexes, in the MS group compared with the NCD group. Of the 30 patients in the MS group, 7 (23.3%) had MVA <1 cm^2^, while 23 (76.7%) had MVA between 1.0 and 1.5 cm^2^, as measured on 2D planimetry. The mean transmitral MDPG was 9.8 ± 5.1 mm Hg.

### Hemodynamic responses to exercise in MS and NCD patients

The hemodynamic parameters obtained via cardiac catheterization are shown in Table 2. At rest, pulmonary pressures were significantly higher in the MS group compared with the NCD group (eg, mean PAWP 19.5 mm Hg vs 9.0 mm Hg, mean PAP 26.0 mm Hg vs 15.0 mm Hg; *P* < 0.001 for both), indicating an elevated resting hemodynamic load. In contrast, resting stroke volume index (47.7 mL/m^2^ vs 50.5 mL/m^2^; *P* = 0.608) and cardiac index (3.4 L/min/m^2^ vs 3.9 L/min/m^2^; *P* = 0.251) did not differ significantly between the 2 groups, nor did resting minute ventilation and arterial O_2_ content.

**Table 2:** Cardiac Catheterization Data at Rest and During Peak Exercise

	At Rest			At Peak Exercise		
	NCD	MS	*P* Value	NCD	MS	*P* Value
Heart rate, beats/min	75.7 ± 12.2	72.6 ± 10.1	0.348	112.2 ± 16.9	116.6 ± 19.7	0.438
Systolic blood pressure, mm Hg	127 ± 21.7	127.6 ± 26.2	0.985	159.8 ± 20.7	158.1 ± 28.1	0.842
Systolic PAP, mm Hg	22.7 ± 4.6	36.9 ± 9.5	**<0.001**	37.9 ± 6.8	73.7 ± 13.1	**<0.001**
Diastolic PAP, mm Hg	9.6 ± 3.4	18.5 ± 5.6	**<0.001**	14.3 ± 4.6	35.7 ± 7.6	**<0.001**
Mean PAP, mm Hg	15.0 ± 3.0	26.0 ± 7.3	**<0.001**	24.9 ± 3.9	52.8 ± 8.7	**<0.001**
Mean PAWP, mm Hg	9.0 ± 2.3	19.5 ± 5.9	**<0.001**	13.8 ± 3.1	39.1 ± 7.0	**<0.001**
Mean right atrial pressure, mm Hg	5.5 ± 2.5	8.1 ± 4.2	**0.010**	8.6 ± 4.5	13.9 ± 5.6	**<0.001**
PVR, Wood units	1.2 ± 0.9	1.3 ± 0.7	0.341	0.7 ± 0.3	1.6 ± 0.7	**<0.001**
PAC, mL/mm Hg	7.5 ± 4.2	4.5 ± 1.7	**0.001**	7.2 ± 3.6	2.3 ± 1.1	**<0.001**
Stroke volume index, mL/m^2^	50.5 ± 19.3	47.7 ± 16.4	0.608	86.8 ± 39.7	49.8 ± 15.2	**<0.001**
Cardiac index, L/min/m^2^	3.9 ± 1.5	3.4 ± 0.9	0.251	9.4 ± 3.3	5.7 ± 1.7	**<0.001**
Arterial O_2_ content, mL/dL	18.7 ± 1.8	18.0 ± 1.6	0.160	18.6 ± 1.4	17.9 ± 1.7	0.156
Venous O_2_ content, mL/dL	14.7 ± 1.9	13.5 ± 1.7	**0.031**	11.5 ± 2.3	8.3 ± 2.0	**<0.001**
Arterial-venous O_2_ difference, mL/dL	3.8 ± 0.8	4.5 ± 0.9	**0.007**	7.1 ± 2.1	9.6 ± 2.1	**<0.001**
Vo_2_, mL/kg/min	3.7 ± 1.1	3.8 ± 0.9	0.374	16.5 ± 4.0	14.2 ± 4.8	**0.027**
O_2_ pulse, mL/beat	3.3 ± 0.9	3.2 ± 1.0	0.734	10.0 ± 2.4	7.4 ± 2.6	**<0.001**
Ventilation, L/min	7.4 ± 1.5	7.0 ± 2.0	0.583	34.4 ± 11.8	29.9 ± 11.4	0.254
Ve/Vco_2_ ratio	38.6 ± 6.2	39.4 ± 6.4	0.642	33.0 ± 5.6	36.8 ± 7.1	**0.005**
LVEDP, mm Hg	NA	13.6 ± 5.1	NA	NA	18.5 ± 6.7	NA
Transmitral MDPG, mm Hg	NA	9.2 ± 4.7	NA	NA	24.1 ± 7.3	NA

Values are mean ± SD. *P* values in **bold** denote statistical significance.

LVEDP = left ventricular end-diastolic pressure; NA = not available; PAC = pulmonary artery compliance; PAP = pulmonary artery pressure; PAWP = pulmonary artery wedge pressure; PVR = pulmonary vascular resistance; Vco_2_ = carbon dioxide consumption; Ve = minute ventilation; Vo_2_ = oxygen consumption; other abbreviations as in Table 1.

**Figure 1:**
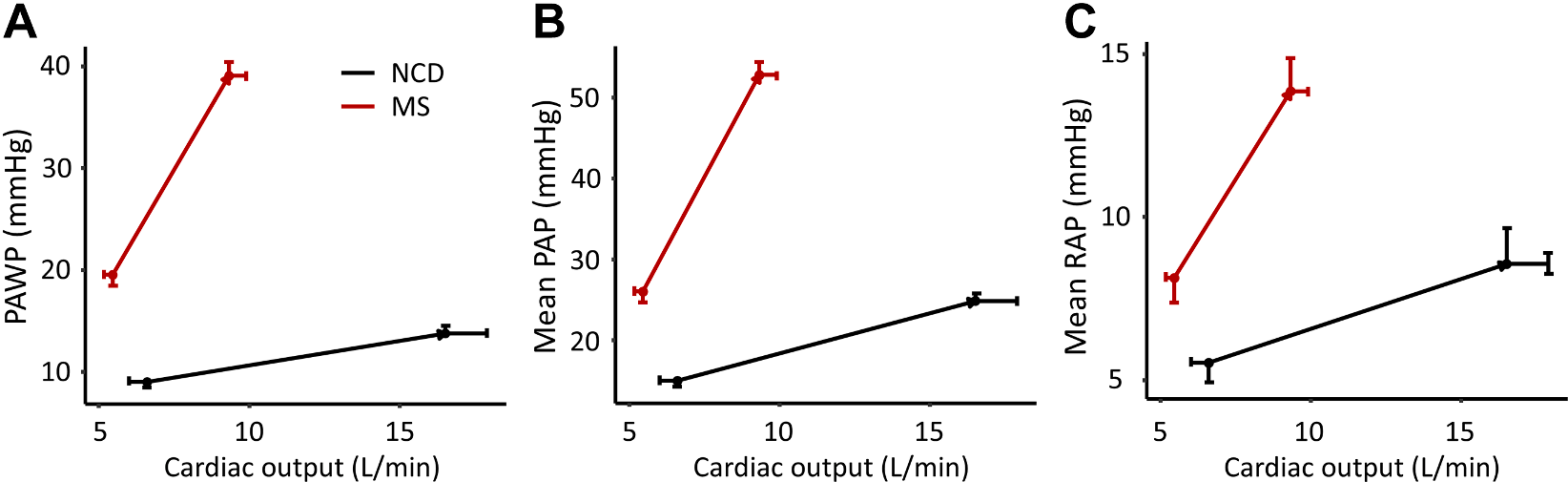
Pressure Slopes in MS and NCD Increases in pulmonary artery wedge pressure (A), mean pulmonary artery pressure (B), and mean right atrial pressure (C) in response to rising cardiac output. MS = mitral stenosis; NCD = noncardiac dyspnea; PAP = pulmonary artery pressure; PAWP = pulmonary artery wedge pressure; RAP = right atrial pressure.

**Figure 2:**
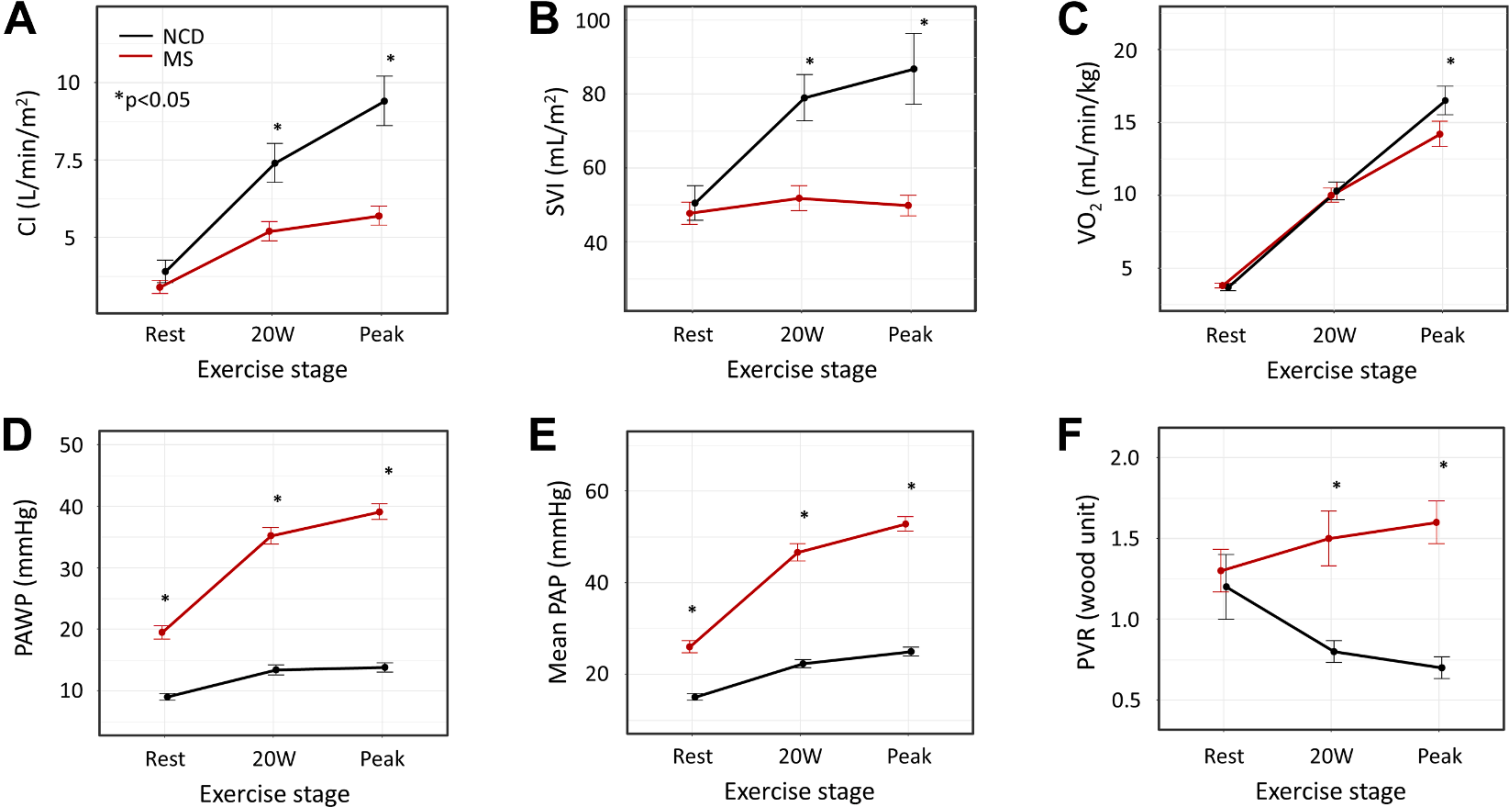
Hemodynamic Changes During Exercise Between MS and NCD Changes in cardiac index (CI) (A), stroke volume index (SVI) (B), oxygen consumption (Vo_2_) (C), PAWP (D), mean PAP (E), and pulmonary vascular resistance (PVR). ∗*P* < 0.05 between MS and NCD. Abbreviations as in Figure 1.

**Figure 3:**
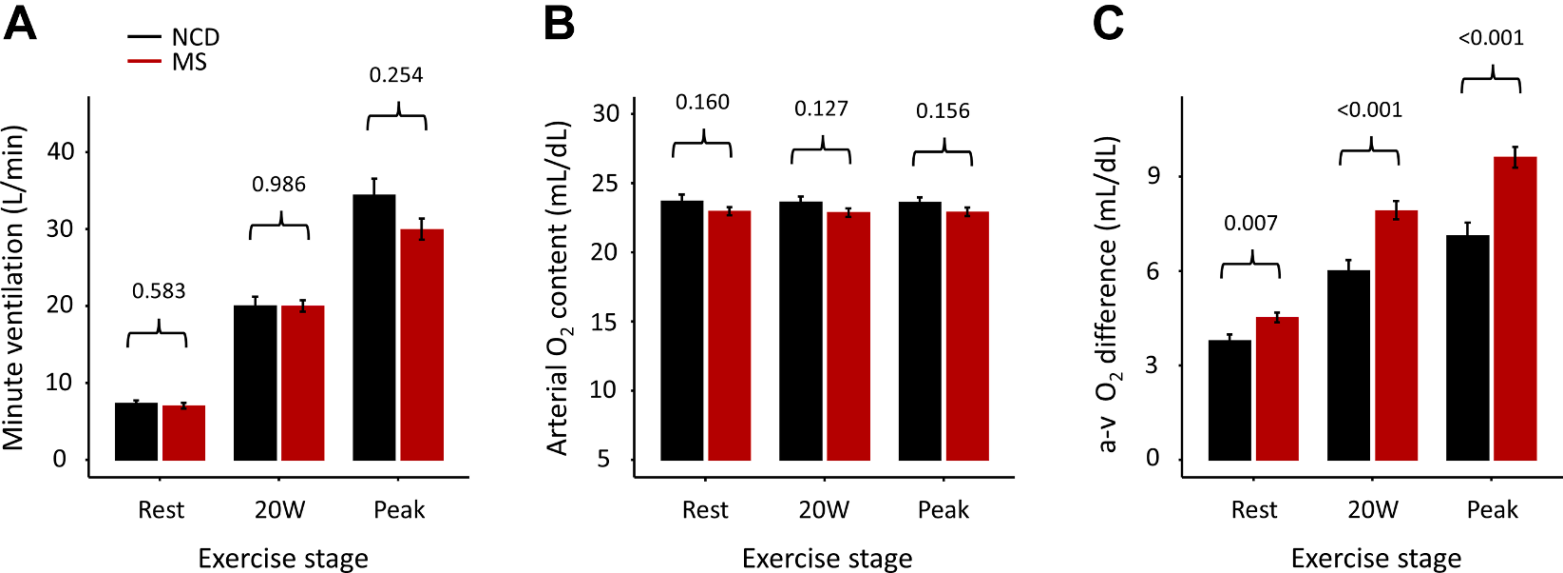
Changes in Minute Ventilation and Oxygen Content During Exercise There were no significant differences in minute ventilation (A) and arterial oxygen content (B) between patients with MS (red) and those with NCD (black). However, patients with MS had a significantly higher arteriovenous (a-v) oxygen difference (C) at rest, 20 W, and peak exercise compared with those with NCD. Abbreviations as in Figure 1.

At peak exercise, MS patients showed a steep rise in filling pressures relative to flow: their PAWP/CO slope averaged 7.6 ± 6.5 mm Hg/L/min (vs 0.6 ± 0.5 mm Hg/L/min in the NCD group), and their mean PAP/CO slope was 10.5 ± 9.0 mm Hg/L/min (vs 1.2 ± 0.6 mm Hg/L/min in the NCD group) (Figure 1). Mean PAWP and PAP at peak exercise were 39.1 ± 7.0 mm Hg and 52.8 ± 8.7 mm Hg in the MS group, compared with 13.8 ± 3.1 mm Hg and 24.9 ± 3.9 mm Hg in the NCD group. MS patients failed to augment stroke volume during exercise, so their peak cardiac index was significantly lower than that of NCD (MS vs NCD, 5.7 ± 1.7 L/min/m^2^ vs 9.4 ± 3.3 L/min/m^2^; *P* < 0.001) (Figure 2). Stroke volume at peak in the MS group was essentially unchanged from rest, whereas it increased markedly in the NCD group. Consequently, exercise capacity was significantly lower in the MS group (peak Vo_2_ 14.2 mL/kg/min vs 16.5 mL/kg/min; *P* = 0.027). Minute ventilation and arterial oxygen content did not differ significantly between the 2 groups; however, the arteriovenous oxygen difference was significantly higher in the MS group at both rest (4.5 mL/dL vs 3.8 mL/dL) and peak exercise (9.6 mL/dL vs 7.1 mL/dL), driven by lower venous oxygen content (Figure 3).

### Parameters associated with exercise capacity in patients with MS

**Figure 4:**
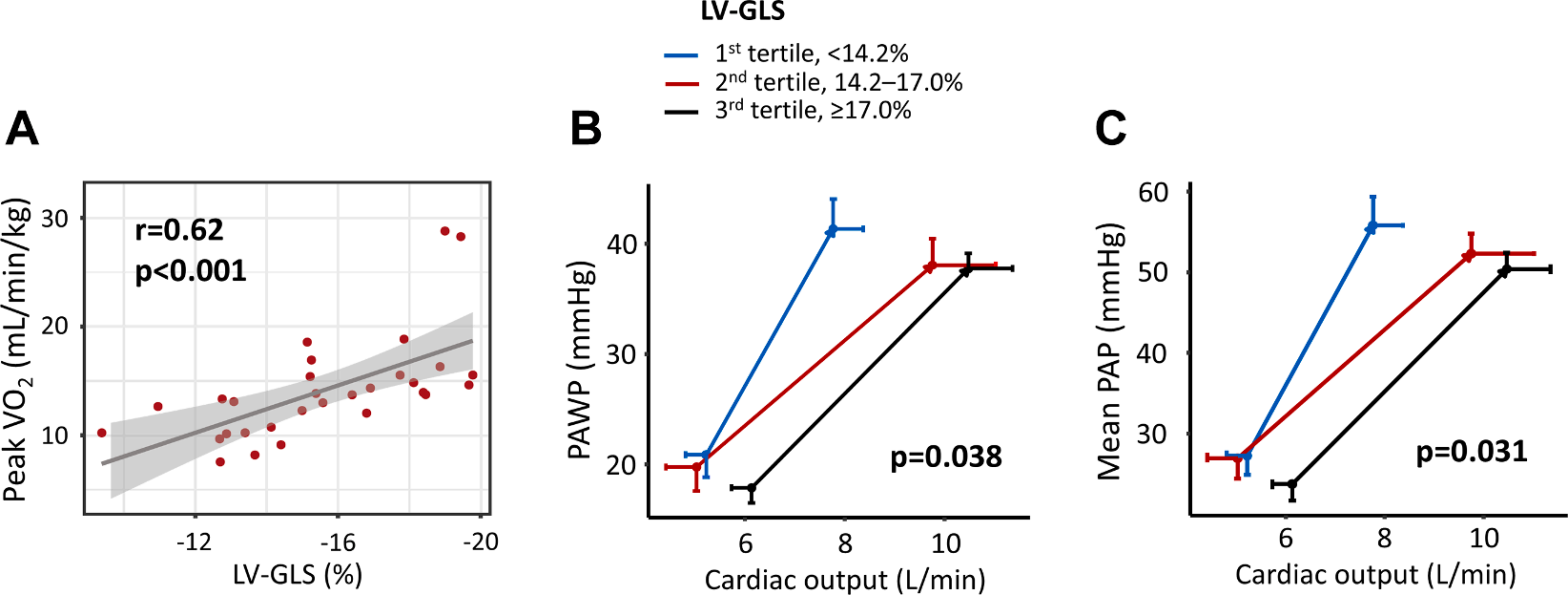
LV GLS and Exercise Hemodynamic Status in Mitral Stenosis Left ventricular (LV) global longitudinal strain (GLS) was significantly correlated with peak Vo_2_ (A), with regression line shown with 95% confidence bands. Patients stratified into tertiles according to LV GLS exhibited different PAWP/cardiac output (CO) slopes (B) and mean PAP/CO slopes (C). Abbreviations as in Figures 1 and 2.

In the MS group, univariable analysis showed that higher resting stroke volume index (*r* = 0.41; *P* = 0.023), higher resting cardiac index (*r* = 0.50; *P* = 0.005), and larger CE MVA (*r* = 0.38; *P* = 0.040) were correlated with higher peak Vo_2_ (Table 3). In contrast, 2D MVA, PHT MVA, and resting transmitral MDPG did not show significant correlations with peak Vo_2_. LV GLS demonstrated a moderate correlation with peak Vo_2_ (*r* = 0.62; 95% CI: 0.34-0.80; *P* < 0.001) (Figure 4A), whereas LA strain did not. Higher absolute LV GLS was associated with greater exercise capacity, and this association remained significant (β = 1.30; 95% CI: 0.80-1.81; *P* < 0.001) after adjustment for sex and atrial fibrillation. Among invasive resting hemodynamic parameters, higher resting PAWP was correlated with lower peak Vo_2_ (*r* = −0.38; *P* = 0.040).

**Table 3:** Determinants of Peak Vo_2_ in Patients With Mitral Stenosis

	β Coefficient	Standardized β	Correlation Coefficient	*P* Value
At rest				
Heart rate			0.10	0.594
Atrial fibrillation			0.05	0.780
Ventilation			0.22	0.825
Echocardiography				
2D MVA			0.26	0.167
PHT MVA			0.10	0.603
CE MVA	6.39	0.38	0.38	**0.040**
Stroke volume index	0.21	0.41	0.41	**0.023**
Cardiac index	3.41	0.50	0.50	**0.005**
Transmitral MDPG			0.30	0.134
Medial e′ velocity			<0.01	0.992
RVSP			0.27	0.145
LA volume index			0.02	0.916
LA reservoir strain			0.15	0.431
|LV GLS|	1.09	0.62	0.62	**<0.001**
Cardiac catheterization				
Cardiac index			0.31	0.094
LVEDP			0.12	0.537
Transmitral MDPG			0.27	0.145
Mean PAWP	-0.31	-0.38	-0.38	**0.040**
Systolic PAP			0.26	0.164
Mean PAP			0.27	0.144
RAP			0.03	0.866
PVR			0.08	0.686
PAC			0.23	0.225

*P* values in **bold** denote statistical significance.

GLS = global longitudinal strain; LV = left ventricular; other abbreviations as in Tables 1 and 2.

Notably, peak Vo_2_ and peak hemodynamic responses were not significantly different among MS patients with differing symptom severity (NYHA functional class I vs II vs III; *P* = 0.827 for peak Vo_2_; *P* = 0.848 for PAWP/CO slope) (Supplemental Table 1), underscoring the limited association between subjective symptoms and objective exercise performance.

### LV GLS and exercise hemodynamic status

Correlations between LV GLS and exercise hemodynamic parameters are presented in Supplemental Table 2. Patients with better LV GLS tended to exhibit more favorable exercise hemodynamic status. Specifically, higher LV GLS correlated with greater peak cardiac index (*r* = 0.50; *P* = 0.005), lower peak systolic PAP (*r* = 0.37; *P* = 0.047), lower pulmonary vascular resistance (*r* = 0.42; *P* = 0.020), and higher pulmonary arterial compliance (*r* = 0.57; *P* = 0.001). These patients also achieved higher oxygen pulse and ventilation at peak exercise, in line with enhanced stroke volume and aerobic capacity (higher peak Vo_2_; *r* = 0.62; *P* < 0.001). Stratification by LV GLS tertiles revealed distinct PAWP/CO and mean PAP/CO slopes during exercise, with lower LV GLS associated with steeper pressure increases (Figures 4B and 4C).

## Discussion

In this prospective mechanistic study, we found a distinct hemodynamic response to exercise in patients with MS requiring intervention by current guidelines. Compared with those with NCD, patients with significant MS exhibited a steep rise in pressure profiles during exercise, without a corresponding increase in stroke volume. A compensatory rise in the arteriovenous oxygen difference was observed, reflecting inadequate CO. Notably, conventional parameters—such as 2D MVA, transmitral MDPG, and even symptom severity—showed no significant correlation with exercise capacity, whereas markers of pump function (stroke volume, LV GLS) and a flow-adjusted valve area (CE-derived MVA) were correlated with exercise performance in MS patients. This study provides valuable insights into the hemodynamic response to exercise in patients with significant MS and identifies predictors of exercise intolerance beyond conventional grading parameters (Central Illustration).

**Central Illustration:**
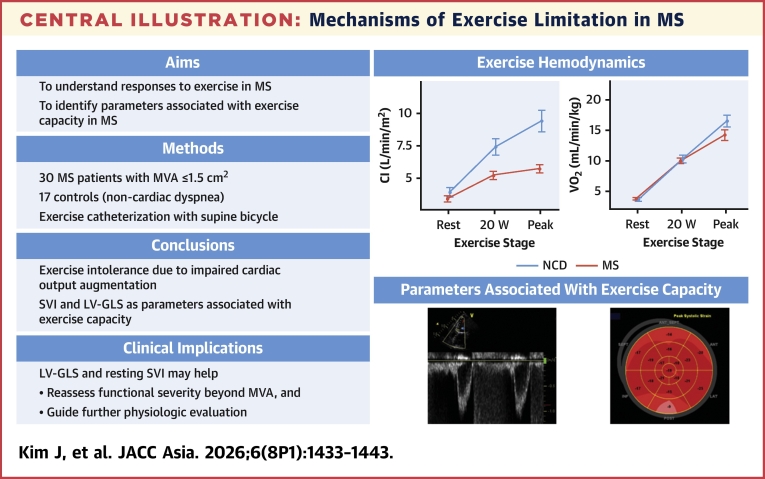
Mechanisms of Exercise Limitation in MS Exercise capacity was assessed using exercise catheterization and cardiopulmonary exercise testing in patients with mitral stenosis (MS), and its relationship with resting echocardiographic and hemodynamic parameters was evaluated, with patients with noncardiac dyspnea (NCD) as a comparator. Limited cardiac output reserve emerged as a key contributor to impaired exercise performance. Resting stroke volume index (SVI) and left ventricular (LV) global longitudinal strain (GLS) showed significant correlations with peak oxygen consumption (Vo_2_). These findings suggest that mitral valve area (MVA) alone may be insufficient to estimate functional capacity, and that an integrated assessment using hemodynamic and imaging parameters is required. ANT = anterior; CI = cardiac index; INF = inferior; LAT = lateral; SEPT = septal.

### Impaired stroke volume augmentation in MS

Stenotic valves limit the increase in blood flow that normally occurs during physical activity, leading to suboptimal LV preload. Accordingly, previous studies have demonstrated an inadequate rise in CO during exercise in patients with MS.^2^^,^^15^ In contrast to prior studies,^2^ we used the direct Fick method to accurately measure stroke volume and CO, rather than Doppler echocardiography, because of concerns regarding image quality during exercise caused by supine position and augmented respiration. In the MS group, stroke volume at peak exercise remained similar to that at rest, unlike the approximately 70% increase observed in the NCD group, resulting in CO that was dependent almost entirely on heart rate. These findings are consistent with previous studies showing little benefit or even potential harm of beta-blocker therapy on exercise capacity, despite its ability to reduce the transmitral pressure gradient in patients with MS.^16^^,^^17^ The significant flow limitations observed in our study population support the current practice guideline recommending valve intervention for patients with MVA <1.5 cm^2^ and symptoms.^5^^,^^6^ Future studies are needed to investigate hemodynamic changes in patients with an MVA of 1.5 cm^2^ or greater.

### Adaptation to insufficient CO

Despite the limited increase in CO, patients with MS exhibited similar Vo_2_ during the early phase of exercise (20 W). Blood gas analysis demonstrated a marked decrease in venous oxygen content, resulting in a higher arteriovenous oxygen difference in patients with MS compared with NCD, indicating a peripheral compensatory response to inadequate CO. Previous studies have shown that patients with MV disease tend to increase oxygen extraction as a compensatory mechanism for impaired oxygen delivery during exercise.^2^^,^^18^^,^^19^ Consistent with this, the MS group in our study showed a greater increase in arteriovenous oxygen difference from rest to peak exercise. However, despite this compensation, the MS group exhibited significantly lower Vo_2_ and oxygen pulse at peak exercise compared with the NCD group.

### Parameters associated with exercise capacity in MS

Previous exercise studies have shown that maximal CO and maximal arteriovenous oxygen difference correlated with exercise capacity, whereas traditional MS severity parameters, such as MVA and the transmitral pressure gradient, did not.^15^^,^^20^ A recent study by Laufer-Perl et al reported similar findings: only peak heart rate response, tidal volume response during exercise, and peak arteriovenous oxygen difference were associated with exercise capacity, whereas MS-related parameters were not.^2^ Consequently, no resting parameters have been useful for predicting exercise tolerance in MS patients to date. Peak Vo_2_ is determined by CO and arteriovenous oxygen difference during exercise. In MS, as stroke volume fails to increase with exercise, a patient’s resting stroke volume effectively represents their maximum forward output. Accordingly, a higher resting stroke volume predicts better exercise capacity (higher peak Vo_2_). Similarly, peak heart rate, representing the other determinant of maximal CO, would be expected to influence exercise tolerance. Consistent with previous studies,^2^ conventional parameters, including MVA assessed using both 2D planimetry and PHT and the transmitral pressure gradient, showed no correlation with exercise capacity. In contrast, MVA calculated using the CE, which incorporates stroke volume, was a significant predictor of exercise capacity.

### Role of LV GLS

GLS has been established as a sensitive marker of myocardial disease and can be impaired even in the subclinical stages of LV dysfunction. LV GLS correlates independently with peak Vo_2_ in patients with heart failure with preserved ejection fraction.^21^ In patients with MS, LV diastolic filling is interrupted and delayed, potentially masking underlying diastolic dysfunction. Several studies have demonstrated that LV GLS is reduced in patients with MS; however, its clinical implications, especially regarding exercise intolerance, remain unknown.^22^ To the best of our knowledge, this is the first study to demonstrate a significant correlation between LV GLS and exercise tolerance in patients with MS. Moreover, higher LV GLS values were associated with greater stroke volume and CO, lower PAPs, and a lower PAWP/CO slope, indicating preserved hemodynamic adaptation during exercise. These findings suggest that LV GLS may serve as a valuable tool for unmasking diastolic dysfunction and predicting exercise tolerance in patients with MS. Importantly, patients with MS who exhibit reduced exercise capacity may also have concomitant myocardial dysfunction resembling a heart failure with preserved ejection fraction phenotype, which can be difficult to distinguish from valvular limitation alone in clinical practice. In this context, LV GLS may provide additional insight into myocardial involvement and help identify patients in whom exercise intolerance reflects not only valvular obstruction but also concomitant myocardial dysfunction. In contrast, LA reservoir strain was not significantly associated with exercise capacity. In severe MS, LA function is uniformly impaired, as reflected by consistently low LA strain values across patients, which likely attenuated its utility to discriminative utility. By comparison, LA strain has demonstrated predictive value in heart failure with preserved ejection fraction and even in mild MS,^23^ in which a broader range of LA function exists.

### Clinical implications

Currently, in patients with MVA <1.5 cm^2^, the presence of symptoms is a major determinant for intervention. However, in our study, no clear association was observed between symptoms and exercise intolerance. Instead, exercise capacity may be influenced by intrinsic patient factors and adaptative mechanisms. Therefore, when planning valvular interventions for patients with significant MS, it may be important to incorporate hemodynamic factors such as stroke volume and chronotropic response, as well as functional echocardiographic parameters such as LV GLS, in addition to anatomical stenosis. Because speckle-tracking analysis is widely available in contemporary echocardiography, LV GLS can be incorporated into routine echocardiographic assessment in patients with MS without requiring additional imaging. In clinical practice, assessment of LV GLS may help identify patients with impaired CO reserve who are more likely to have limited exercise capacity.

### Study limitations

First, the small sample size limits the interpretation and generalizability of our findings. Although conventional parameters, including symptom severity, did not show a significant correlation with peak Vo_2_, larger studies are needed to confirm these results. Given the limited sample size and the absence of atrial fibrillation in the NCD group, comprehensive multivariable adjustment for baseline differences such as sex distribution and atrial fibrillation between groups was limited. In addition, given the exploratory nature of this study and the number of comparisons performed, no formal adjustment for multiple testing was applied. Therefore, the findings, particularly secondary correlations, should be interpreted as hypothesis generating. Accordingly, the study was not powered to detect small to moderate associations, and nonsignificant findings should be interpreted with caution.

Second, despite the strict criteria to exclude heart failure, the control group may not represent truly healthy volunteers, as they underwent exercise catheterization because of dyspnea or equivalent symptoms. Thus, they likely had lower exercise capacity than healthy individuals, which could underestimate the difference between MS patients and normal subjects.

Third, the use of a supine bicycle test may have promoted volume overload, potentially augmenting pressure profiles during exercise compared with those observed during upright exercise or daily activities.

Fourth, beat-to-beat variation caused by atrial fibrillation in patients with MS may have influenced echocardiographic measurements; however, the main parameters were obtained via catheterization, averaging multiple beats to minimize variability. In addition, atrial fibrillation and repeated measurements across exercise stages may have introduced residual variability that could not be fully accounted for with the current study design. Because of the limited sample size, stratified analyses by rhythm status and more advanced modeling approaches, such as mixed-effects models, were not feasible.

Finally, this was a single-center study conducted in a predominantly rheumatic MS population undergoing supine bicycle exercise testing. Therefore, our findings may have limited applicability to the broader spectrum of patients with MS, particularly those with degenerative etiologies such as mitral annular calcification or older Western populations.

## Conclusions

Patients with MVA <1.5 cm^2^ exhibited limited stroke volume augmentation during exercise, leading to exercise intolerance, along with evidence of peripheral adaptation to inadequate CO. As conventional severity parameters did not significantly correlate with exercise tolerance, these findings highlight the importance of hemodynamic assessments beyond MVA or pressure gradients when evaluating patients with significant MS. In addition, LV GLS emerged as a valuable parameter for predicting exercise tolerance and hemodynamic responses during exercise in this population.

## Funding Support and Author Disclosures

This research was supported by a grant from the Korean Society of Echocardiography, Republic of Korea (PHO0232471), and the National Research Foundation of Korea grant funded by the Korean government (RS-2022-NR069463). The funders had no role in the design and conduct of the study; collection, management, analysis, and interpretation of the data; preparation, review, or approval of the manuscript; and decision to submit the manuscript for publication. The authors have reported that they have no relationships relevant to the contents of this paper to disclose.
