# Supplementary material for: Hemodynamic Responses to Exercise and Predictors of Exercise Capacity in Mitral Stenosis: Exercise Catheterization Study
Source: JACC Asia. 2026 Jun 25;6(8):1433–43. doi: 10.1016/j.jacasi.2026.04.040 (PMC13458926; doi:10.1016/j.jacasi.2026.04.040)
Supplement: Supplementary file 1 [file mmc1.docx]

**Hemodynamic Responses to Exercise and Predictors of Exercise Capacity in Mitral Stenosis: Exercise Catheterization Study**

**Supplementary Table 1. Exercise hemodynamics according to symptom severity in patients with mitral stenosis**

| **Variable** | **NYHA Fc I (n=5)** | **NYHA Fc II (n=15)** | **NYHA Fc III (n=10)** | **P value** |
| --- | --- | --- | --- | --- |
| Age | 59.4 ± 3.8 | 57.3 ± 10.2 | 55.5 ± 9.2 | 0.735 |
| Male | 2 (40.0) | 3 (20.0) | 2 (20.0) | 0.628 |
| NT-proBNP, pg/mL | 355.8 ± 368.0 | 584.6 ± 547.8 | 653.5 ± 668.5 | 0.544 |
| 2D-MVA | 1.2 ± 0.2 | 1.1 ± 0.1 | 1.1 ± 0.3 | 0.808 |
| Trans-mitral MDPG, mmHg | 7.8 ± 2.3 | 10.7 ± 6.1 | 9.4 ± 4.5 | 0.424 |
| At peak exercise |  |  |  | 0.706 |
| Cardiac index, L/min | 5.7 ± 1.6 | 5.7 ± 1.9 | 5.7 ± 1.7 | 0.994 |
| VO_2_, mL/kg/min | 16.3 ± 7.1 | 14.0 ± 5.0 | 13.5 ± 3.3 | 0.827 |
| Trans-mitral MDPG^*^, mmHg | 22.6 ± 6.2 | 22.9 ± 7.5 | 20.1 ± 6.7 | 0.395 |
| PAWP, mmHg | 39.8 ± 9.1 | 38.8 ± 6.1 | 39.2 ± 7.9 | 0.964 |
| PAWP/CO slope | 7.3 ± 3.5 | 6.2 ± 3.2 | 9.8 ± 10.4 | 0.848 |

Data are presented as mean ± standard deviation or n (%).

CO, cardiac output; MDPG, mean diastolic pressure gradient; 2D-MVA, two-dimensional mitral valve area; NT-proBNP, N-terminal pro–B-type natriuretic peptide; NYHA, New York Heart Association; PAWP, pulmonary artery wedge pressure; VO_2_, oxygen consumption.

**Supplementary Table 2. Correlations between LV-GLS and hemodynamic parameters in patients with mitral stenosis**

| **Variable** | **β coefficient** | **Correlation coefficient** | **P value** |
| --- | --- | --- | --- |
| **At peak exercise** |  |  |  |
| Cardiac index, L/min/m^2^ | 0.32 | 0.50 | **0.005** |
| LVEDP, mmHg |  | 0.04 | 0.818 |
| Trans-mitral MDPG by cath, mmHg |  | <0.01 | 0.094 |
| Mean PAWP, mmHg |  | 0.18 | 0.354 |
| Systolic PAP, mmHg | -1.74 | -0.37 | **0.047** |
| Mean PAP, mmHg |  | 0.23 | 0.229 |
| Mean right atrial pressure, mmHg |  | 0.12 | 0.547 |
| PVR, wood unit | -0.11 | -0.42 | **0.020** |
| PAC, mL/mmHg | 0.22 | 0.57 | **0.001** |
| Arterial O_2_ content, mL/dL |  | 0.19 | 0.318 |
| Venous O_2_ content, mL/dL |  | 0.04 | 0.844 |
| a-v O2 difference, mL/dL |  | 0.18 | 0.335 |
| VO_2_, mL/kg/min | 1.09 | 0.62 | **<0.001** |
| O_2_ pulse, mL/beat | 0.46 | 0.49 | **0.006** |
| Ventilation, L/min | 1.51 | 0.36 | **0.048** |
| PAWP/CO slope, mmHg/L | -1.19 | -0.50 | **0.005** |
| Mean PAP/CO slope, mmHg/L | -1.79 | -0.55 | **0.002** |

a-v, arterio-venous; CO, cardiac output; LV, left ventricular; LVEDP, left ventricular end-diastolic pressure; LV-GLS, left ventricular global longitudinal strain; PA(W)P, pulmonary artery (wedge) pressure; PAC, Pulmonary artery compliance; PVR; pulmonary vascular resistance; VO_2_, oxygen consumption.
